# Creating a Global Legal and Policy Database and Document Repository: Challenges and Lessons Learned From the World Health Organization Sexual, Reproductive, Maternal, Newborn, Child and Adolescent Health Policy Survey

**DOI:** 10.34172/ijhpm.2021.153

**Published:** 2021-11-06

**Authors:** Elizabeth Katwan, Geoffrey Bisoborwa, Betzabe Butron-Riveros, Sergei Bychkov, Kwami Dadji, Natalia Fedkina, C. Anoma Jayathilaka, Dhiraj Kumar, Zhao Li, Rajesh Mehta, Neena Raina, Khalid Siddeeg, Laura Ferguson, Line Neerup Handlos, Ashley Sheffel, James Kiarie, Mario R. Festin, Theresa Diaz

**Affiliations:** ^1^Department of Maternal, Newborn, Child and Adolescent Health and Ageing, World Health Organization (WHO), Geneva, Switzerland.; ^2^Universal Health Coverage - Life Course, World Health Organization Regional Office for Africa, Brazzaville, Congo.; ^3^Department of Family, Health Promotion and Life Course, Pan American Health Organization, Washington, DC, USA.; ^4^WHO European Office for Prevention and Control of Noncommunicable Diseases, WHO Regional Office for Europe, Moscow, Russia.; ^5^WHO Country Office for the Democratic Republic of the Congo, Kinshasa, Democratic Republic of the Congo.; ^6^Department of Reproductive, Maternal, Newborn, Child, Adolescent Health and Ageing, WHO Regional Office for South-East Asia, New Delhi, India.; ^7^Division of Health Systems and Services, WHO Regional Office for the Western Pacific, Manila, Philippines.; ^8^Department of Healthier Population, WHO Regional Office for the Eastern Mediterranean, Cairo, Egypt.; ^9^Institute on Inequalities in Global Health, Keck School of Medicine, University of Southern California, Los Angeles, CA, USA.; ^10^Euro Health Group, Copenhagen, Denmark.; ^11^Department of International Health, Johns Hopkins Bloomberg School of Public Health, Baltimore, MD, USA.; ^12^Department of Sexual and Reproductive Health and Research, WHO, Geneva, Switzerland.; ^13^Department of Obstetrics and Gynecology, College of Medicine, University of the Philippines Manila, Manila, Philippines.

**Keywords:** Global Policy Database, Health Policy Survey, Reproductive Health, Maternal and Newborn, Child and Adolescent, Health Policy Database

## Abstract

The World Health Organization (WHO) has collected information on policies on sexual, reproductive, maternal, newborn, child and adolescent health (SRMNCAH) over many years. Creating a global survey that works for every country context is a well-recognized challenge. A comprehensive SRMNCAH policy survey was conducted by WHO from August 2018 through May 2019. WHO regional and country offices coordinated with Ministries of Health and/or national institutions who completed the questionnaire. The survey was completed by 150 of 194 WHO Member States using an online platform that allowed for submission of national source documents. A validation of the responses for selected survey questions against content of the national source documents was conducted for 101 countries (67%) for the first time in the administration of the survey. Data validation draws attention to survey questions that may have been misunderstood or where there was a lot of missing data, but varying methods for validating survey responses against source documents and separate analysis of laws from policies and guidelines may have hindered the overall conclusions of this process. The SRMNCAH policy survey both provided a platform for countries to track their progress in adopting WHO recommendations in national SRMNCAH-related legislation, policies, guidelines and strategies and was used to create a global database and searchable document repository. The outputs of the SRMNCAH policy survey are resources whose importance will be enriched through policy dialogues and wide utilization. Lessons learned from the methodology used for this survey can help to improve future updates and inform similar efforts.

## Background

Under the Sustainable Development Goals and the Global Strategy for Women’s, Children’s and Adolescents’ Health (GSWCAH) – 2016-2030,^1^ countries have committed to improving the health of women, children and adolescents through multi-sectoral action. A 2020 progress report on the GSWCAH found that while there has been progress overall, some key indicators, such as reducing neonatal mortality, are stagnating or even reversing for some areas, such as coverage for childhood immunization.^2^ To accelerate progress and improve in areas that remain stagnant, the adoption and implementation of evidence-informed and equity-focused laws, policies, and guidelines is required.

The World Health Organization (WHO) has collected information on laws and policies on maternal, newborn, child and adolescent health (MNCAH) and on sexual and reproductive health (SRH) for many years. In response to recent calls by countries for consolidation of separate data collection efforts, a comprehensive survey on sexual, reproductive, maternal, newborn, child and adolescent health (SRMNCAH) laws and policies was administered from 2018 to 2019. This survey built upon previous separate efforts and, for the first time, asked for source documents to be submitted, which allowed for responses to be validated against national laws, policies and guidelines. The questionnaire was updated to align with WHO recommendations and global strategies developed since the last enumeration in 2016, providing a platform for tracking country progress in adopting WHO recommendations in national SRMNCAH-related laws, policies, strategies and guidelines and a mechanism for understanding the broader context related to health outcomes under GSWCAH. A report detailing findings from the survey showed that over 80% of surveyed countries have national policies for most key SRMNCAH areas. The survey also found that over 90% of countries have policies on SRH, antenatal care, childbirth, postnatal care, and child health, but fewer have policies on adolescent health and violence against women.^3^

## Methods

###  Process for Development of Survey Tool

To optimize the approach used for SRMNCAH policy tracking, WHO established an SRMNCAH policy reference group to obtain external expert advice on the contents of the survey. The group’s members, via an online survey, identified priority areas within SRMNCAH to include in the survey and suggested topics that could be excluded. Concurrently, WHO researched existing global policy and legislative databases and found thirty SRMNCAH-related data sources, identifying key topics that could be eliminated from the SRMNCAH policy survey.^3^

To create a final questionnaire that combined previously separate data collection efforts, key focal points in multiple WHO departments and from all six WHO regional offices, along with stakeholders in partner organizations, reviewed the draft questionnaire and provided feedback on its content. As it was important to provide a common understanding of key technical terms used throughout the survey, a glossary was included in the questionnaire. The survey tool was available in all official United Nations (UN) languages (Arabic, Chinese, English, French, Russian, Spanish) and Portuguese.^4^

Various resources were consulted to determine the definitions used for all terms in the glossary. The definitions provided within the survey for guideline,^5^ health policy,^6^ law^7^ and healthy strategy/plan^8^ (Table 1) help to show the differences and relationships between these key terms. Strategic plans often build off policies in that a health policy provides a vision and outlines goals for health outcomes, while a national health strategy sets forth the process for achieving these. Guidelines similarly work in conjunction with policies and strategic plans, providing evidence-based guidance on various interventions and public health activities for key stakeholders. Additionally, health policies can describe priorities and roles of stakeholders and provide information to a population, while laws “govern behaviour” showing that policies and laws should ideally reflect each other and work in parallel.

**Table 1:** Key Definitions From SRMNCAH Policy Survey Glossary

**Guideline**	“Guidelines are systematically developed evidence-based statements which assist providers, recipients and other stakeholders to make informed decisions about appropriate health interventions. Health interventions are defined broadly to include not only clinical procedures but also public health actions”^5^ (p. 2).
**Health policy**	“Health policy refers to decisions, plans, and actions that are undertaken to achieve specific healthcare goals within a society. An explicit health policy can achieve several things: it defines a vision for the future which in turn helps to establish targets and points of reference for the short and medium term. It outlines priorities and the expected roles of different groups; and it builds consensus and informs people.”^6^
**Law**	“Laws are rules that govern behavior. Laws can be made by a legislature, resulting in primary legislation (often called statutes or acts), by executive or local government through the issue of secondary legislation (including decrees, regulations and bylaws), or by judges through the making of binding legal precedent (normally in common law jurisdictions)”^7^ (p. vii).
**National health strategy **	“National health strategy, also known as a national health strategic plan or national health plan” is “a process of organizing decisions and actions to achieve particular ends, set within a policy, providing ‘a model of an intended future situation and a program of action predetermined to achieve the intended situation.’ Refers to the broad, long term lines of action to achieve the policy vision and goals for the health sector, incorporating ‘the identification of suitable points for intervention, the ways of ensuring the involvement of other sectors, the range of political, social, economic and technical factors, as well as constraints and ways of dealing with them’”^8^ (p. 11).

Abbreviation: SRMNCAH, sexual, reproductive, maternal, newborn, child and adolescent health.

 In previous rounds of the MNCAH policy survey, respondents reported on the existence of national laws, policies, guidelines, and strategies within their countries without providing the source documents from which these responses stemmed. Some of the questions had been asked in a manner that could have led to overly affirmative responses, such as whether a national guideline followed WHO recommendations rather than asking whether the national guideline contained specific interventions which would allow for an assessment of alignment with WHO guidance. For the 2018-2019 SRMNCAH policy survey, use of an online platform for data collection permitted respondents to submit source documents, allowing for validation of responses.

###  Approaches to Data Collection 

 The questionnaire was structured into several modules: Cross-cutting SRMNCAH, Maternal and newborn health, Child health, Adolescent health, Reproductive health, and Gender-based violence. An online platform, programmed using LimeSurvey software, permitted various respondents to be assigned to specific modules, allowing for concurrent data entry within the modules by several users within a single country. Two webinars were held to train regional focal points in how to use the online survey platform. A user guide and video tutorial were also provided.

Regional focal points for the survey coordinated with assigned focal points in each country to collect information on designated respondents. The WHO country office, or other assigned country focal point, was responsible for coordinating with the Ministry of Health (MoH) or national agencies/institutions to complete the survey. In the majority of participating countries, the principal respondents were from the MoH. Multiple respondents may have been consulted on specific topics to assist in completion of the survey, including officials from other government agencies, WHO country offices and other UN agencies. For each module, there was slight variation in the affiliations of personnel who assisted the main MoH respondent in completing the questionnaire, however the majority of those consulted were others within the MoH or from WHO (Figure).

**Figure:**
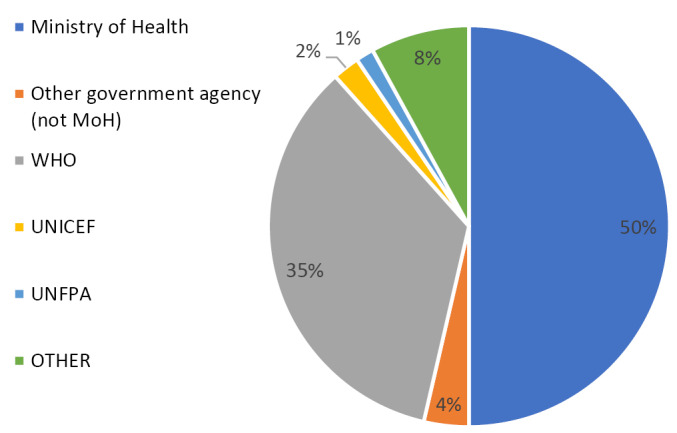
Affiliations of Respondents Consulted by Main Respondent for the Cross-Cutting Module of the Survey. Abbreviation: MoH, Ministry of Health; WHO, World Health Organization; UNFPA, United Nations Population Fund; UNICEF, United Nations Children’s Fund.

 Various approaches to administering the survey were employed by WHO regional offices and country teams based on whether WHO has a country office presence. In countries with WHO country offices, SRMNCAH focal points coordinated with the MoH and partners to complete the survey. In countries without a WHO country office, WHO regional focal points coordinated survey completion directly with the MoH and/or other national institutions. In the Western Pacific Region, after the MoH focal points were identified through coordination with WHO country offices, the regional office focal point provided a brief overview of the survey and monitored progress. Amongst countries within the Region of the Americas, Ministries of Health nominated a survey coordinator to organize inputs from colleagues and to review the responses before submitting. If the MoH requested support, the regional office provided an external professional to interview all relevant units and fill out the survey online.

 The survey was designed with the ability to be filled out by multiple respondents based on their area of expertise, allowing for specific respondents to be mapped to defined modules to complete just those sections. The questionnaire could also be completed offline by multiple respondents with a single respondent entering the responses into the survey platform. A limited number of countries were able to hold review meetings of the completed questionnaire with key stakeholders allowing for agreed revisions before submitting the final version though some countries reviewed the survey responses after submission and requested edits which were incorporated into the database used for final analysis by WHO.

Response rate to the SRMNCAH policy survey varied by region. Of all 194 WHO Member States, 150 completed the survey (77%) (Table 2).^9^ Variation in response rates were due to several reasons.

**Table 2:** Response Rate to 2018-2019 SRMNCAH Policy Survey

**WHO Region**	**Total Number of WHO Member States**	**Number of WHO Member States Participated in Survey**	**Response Rate Among WHO Member States**
African region	47	42	89%
Region of the Americas	35	29	83%
Eastern Mediterranean region	21	15	71%
European region	53	39	74%
South-East Asia region	11	11	100%
Western Pacific region	27	14	52%
**Total**	**194**	**150** ^a^	**77%**

Abbreviations: SRMNCAH, sexual, reproductive, maternal, newborn, child and adolescent health; WHO, World Health Organization. ^a^Five additional non-Member States, which are not reflected in the above response rate, completed the survey. These include: British Virgin Islands (*Americas*), French Polynesia (*Western Pacific*), Guam (*Western Pacific*), Occupied Palestinian territory (*Eastern Mediterranean*), Wallis and Futuna Islands (*Western Pacific*).

 Survey enumeration lasted from August 2018 through May 2019. Several regional offices thought that the timing of the beginning of data collection, in coinciding with various holiday schedules and end of year administrative processes, factored into a low response rate by the end of that year. Due to this, the period of enumeration was extended through May 2019.

 Most regional offices listed staffing issues within each country as a main factor in why some were unable to complete the survey. The main issue cited was limited capacity amongst Ministries of Health to direct time towards completing the survey. Specific issues included staff turnover, dismissals, retirements; changes in leadership and internal restructuring in MoH and/other national institutions; and limited personnel covering the wide range of programs of SRMNCAH. In the Western Pacific Region, staff in a few countries were sometimes diverted to public health emergencies during the time of survey enumeration.

 Commitment from the MoH was another key factor in countries’ completion of the survey. The Regional Office of the Americas stressed the importance of informing countries of the potential benefits of the survey results for planning, updating current policies and strategies, and assessing SRMNCAH programs. In the South-East Asia Region, where all countries completed the survey, obtaining government agreement and identifying a focal point were key factors to this full response rate. The European Regional Office cited lack of official hard copy invitation letters as a possible reason for some Ministries not participating.

 MoH commitment also extended to the final step of authorizing submission of the survey responses. For example, in one country, the survey was completed but the MoH did not authorize its submission, citing that more time was needed to review the survey. Despite follow up from the regional office, the survey was not submitted.

 Connectivity issues were also a challenge across several regions, due to low bandwidth, outdated software, and high security settings that blocked access to the survey platform. Some regional and country offices offered to enter the survey responses into the online platform in these situations. Two countries within the African and Eastern Mediterranean regions had issues in submitting their completed surveys online and were unable to submit them within the deadline.

###  Cataloguing National Source Documents Into a Searchable Repository

 Participating countries submitted national source documents used to support their answers to the survey. For each document, country respondents were asked to provide information on the type of document, year of publication and language when uploading the document.

 The cataloguing process involved several activities: standardizing file names; categorizing documents by key identifying characteristics; and creating a database which linked each document to the survey question(s) for which they were uploaded to be used as a key for validating survey responses against the source documents.

 Standardizing file names for the online repository was completed simultaneously with the larger task of cataloguing the documents based on key characteristics. Each document was reviewed to confirm its official title, topic, type, year of publication, and language as the initial categorization. Many documents fell under multiple topics, for example covering both child and adolescent health. It was also necessary to re-categorize some documents from the initial survey categorization. Despite providing definitions of the various types, many of the documents were mis-categorized, indicating that some respondents may not have been able to clearly distinguish between a law, policy, guideline, or strategic plan.

 The end result of the cataloguing process was the creation of a searchable repository of national laws, policies, guidelines, and strategic plans related to SRMNCAH accessible to the public. This repository allows for analysis of the content of the documents as well as validation of the survey responses against the source documents completed in 2020.

###  Validation of the Survey Responses

 Validation of the survey responses against the source documents was completed in 2020. The work was split between two institutes, one focusing on laws and one focusing on policies, guidelines and operational guidance. Each institute applied their expertise to reviewing the preliminarily catalogued documents to ensure that they were correctly categorized and to abstracting information from the documents to verify the responses to specified questionnaire items. A set of questionnaire items were selected from the full survey tool for inclusion in the validation exercise. For validation of laws, only documents in one of the six official UN language were included in the analysis. For validation of policies, guidelines, and operational guidance, in addition to the UN languages, documents in Danish, Serbo-Croatian, and Slovenian were included. This reduced the dataset for validation from the 150 WHO Member States that completed the survey to 99 countries for validation of laws and 101 countries for validation of policies, guidelines and operational guidance documents. Within both institutes, research assistants with qualifications in the respective languages conducted the validation. Study protocols were followed and all research assistants received training prior to starting the work. Project managers conducted regular quality checks.

 WHO provided the institutes with a database of the survey responses and a database of the source documents, mapping which documents were uploaded for specific content sections of the questionnaire. From the selected set of questions for validation, the institutes searched the documents for the relevant content necessary to check whether the responses were congruent with the text. This included searching the documents for selected key words or phrases specific to each survey question. As a first step, the document(s) specifically used to answer each survey section were searched. When a discrepancy was discovered, details documenting it were recorded. Confirmation of the survey responses as well as instances when no information was available in the document(s) to support the survey response were also recorded, hence, the following categories were used to classify the findings:

- Match – Survey response was consistent with or supported by information in the source document;
- Mismatch – survey response contradicted information in the source document, or;
- Unable to be validated
  ♦ Information not available in document - No relevant information was available in the source document that was uploaded for that question, or;
  ♦ Document not available - No source document had been uploaded to support the answer or no source document in UN language available.

Overall, of the 101 countries included in the validation of responses against policy, guideline, and operational guidance documents, a quarter of the validated survey responses (26%) corresponded to information found in the source documents (matches). Four percent of the responses contradicted information found in the source document (mismatches), whereas 53% of the responses could not be validated either because no document on the topic was uploaded (37%), or because the relevant information was not available in the document uploaded for the question (16%).

 For validation of responses against laws, 99 countries were included in the analysis. Fourteen percent of the validated survey responses corresponded to information found in the documents provided (matches). An inconsistency in response was found in 13% of responses (mismatches), due to information in the documents contradicting the responses or no information being found in the documents provided. Seventy-three percent of responses were unable to be validated primarily because no law documents were uploaded under that question; but also in cases where no law documents were submitted for that question in a UN language.

## Discussion

###  Strengths, Limitations and Lessons Learned

 The 2018-2019 enumeration of the SRMNCAH policy survey presented both challenges and successes at all stages, from development of the questionnaire and administration of the survey to validation of the responses against source documents. Rather than focusing only on the successes of the work, it is important to recognize the challenges inherent in this type of complex data collection and to share lessons learnt in facing these challenges with the hope this can help inform future efforts in this area.

 While the purpose of combining previously separate efforts in monitoring laws and policies for MNCAH and for SRH was to streamline data collection activities within WHO Member States, combining content greatly expanded the survey tool. Despite an attempt to limit the burden of data collection, through conducting a review of policy and legal databases to determine areas that, while important to SRMNCAH, already had available information and thus did not need to be collected through this exercise, the final questionnaire was very long and complex. Additionally, since the survey tool changed significantly from previous rounds and few countries participated in all survey rounds, limited trend analyses are possible.

 During enumeration of the survey, a key to success was the regular communication between WHO regional and country offices with MoH officials. Timely responses to respondent queries and regular follow-up were crucial to successful administration of the survey. Several regional offices stressed the importance of providing the background and purpose of the survey as well as the possible uses of the data in getting buy-in from the MoH. Focal points from the Western Pacific Regional Office communicated to countries that the survey would generate an open global database for the country’s reference. In the Eastern Mediterranean region, similar engagement led to the survey being perceived by many countries as a timely and comprehensive exercise.

 Interpretation of the questions, was a limitation of the survey in all languages. Beyond questions of the accuracy of the translation of the questionnaire, raised by WHO regional offices for the Region of Americas and the European Region, knowledge of the nuances between laws, policies, guidelines, and strategic plans, left room for respondents to interpret the questions differently. While technical experts were assigned for each survey module by the MoH and a glossary of key terms was provided, responses could vary based on the respondent’s knowledge of the national SRMNCAH policy landscape. Additionally, survey questions were framed towards national policies, so countries with a decentralized health system, which may also have decentralized policies, found it difficult to respond to some questions. Better capturing of the policy landscape in countries with decentralized health systems should be considered in future rounds of this survey. Finally, the survey purposely does not address implementation of policies, as this can be difficult to accurately assess. Therefore, findings must be understood to represent the legal and policy environment as it exists on paper with limited insight into the degree to which these are implemented.

 An online platform was used for the first time during the 2018-2019 policy survey, which allowed several opportunities to improve the enumeration. Past rounds of the policy survey were done using Microsoft Excel with no built-in skip patterns; using LimeSurvey software allowed for programming of skip patterns, creating mandatory data entry fields, and providing concurrent data entry capacity between the different survey modules. The Regional Office for South-East Asia noted that the policy survey served as capacity building for MoH and WHO country office staff in online survey administration.

 However, there were some issues with the online platform and, despite testing, there were complications in using it widely for the first time. Given the length and diversity of question types in the survey, the native features of LimeSurvey required heavy adaptation which, in some cases, led to information loss. Application of skip patterns from the questionnaire was not always consistently programmed across modules and while the platform allowed for concurrent data entry between the survey modules, this meant that users were able to skip between modules, which did not activate the mandatory field checks. Several country teams felt restricted by the mandatory fields though, noting that none of the response choices reflected their situation, however they were required to provide a response. Finally, once the survey was submitted, users could no longer edit their responses, which prevented them from making further revisions directly. Many of these challenges might be overcome in future survey rounds with additional pilot testing of the survey tool, fewer or simpler questions and different rules around mandatory fields.

One major advantage of the online platform was the facility to upload source documents which were used to build a searchable repository of documents. Once publicly available through the WHO MNCAH data portal,^10^ this repository, which contains all documents submitted by national respondents, will serve as a global resource for anyone interested in a better understanding of SRMNCAH-related legal and policy environments. These documents were also used to validate responses to selected questionnaire items, which is a significant improvement upon past iterations of the survey where this was not possible. This may help to address some data quality issues or provide additional information regarding the content of a national law, policy, or guideline if not directly asked in the survey. This exercise may also provide an example of a methodology to extract information directly from source documents rather than enumerating a full survey in the future.

 The validation of survey responses against source documents also had limitations. One key constraint of the exercise was that it was limited to uploaded law, policy, guideline, and operational guidance documents, but not other documents referenced to complete the survey, such as strategic plans. Also, only documents in a limited set of languages were reviewed. It cannot be guaranteed that information relevant to the validation of the survey responses did not appear in other document types or documents in other languages. Furthermore, as the validation was done by two institutes based on their areas of expertise, there were minor differences in their protocol, such as the way they categorized mismatched findings, and there was no joint analysis of the results. This remains an outstanding step for complete validation.

 Based on the results of the validation of policies and guidelines, eight of the ten questions with the most mismatching responses in the questionnaire came from the maternal and newborn health module. For example, survey responses on the timing of postnatal care contacts did not match the information in the source documents, suggesting that these questions were either difficult to understand or that the respondents were only familiar with the content of these policies/guidelines to a limited degree. With regard to laws, the highest number of mismatching responses were found in the cross-cutting module. For example, survey responses on the existence of a legal requirement for training of health workers in filling out death certificates using the International Classification of Diseases appears to have been difficult for countries to answer. Examining the phrasing of the questions that were found to have the most mismatched responses is an important finding to try to improve the questionnaire in future rounds of the survey.

###  Next Steps

 The SRMNCAH policy survey has provided a wealth of information that will guide both immediate and forthcoming next steps.

 The findings from the validation exercise suggest that it might be possible to update the policy survey based on abstraction from source documents with confirmation from an MoH. Requesting respondents to upload source documents rather than responding to a questionnaire, and then having a team with relevant content knowledge review and extract the information from the documents might result in more precise survey responses and more detail on the content, while reducing the burden on country teams to complete a long questionnaire. Conversely, this method is resource intensive at the central level and it may be not feasible to abstract information from documents in all available languages. This method may also not facilitate country engagement or ownership of the survey results, which should ideally serve to improve understanding of SRMNCAH-related laws and policies in-country and generate interest about using this information to improve national responses within these areas of health.

 The outputs of the SRMNCAH policy survey, both questionnaire responses and the repository of national documents, are a resource whose importance will be enriched through wide utilization. The data have already been used in various regional activities. In the African region, results of the survey were presented and discussed at an annual RMNCAH Review and planning meeting involving SRMNCAH focal points from 36 out of the 47 WHO country offices. The survey has provided the WHO African Region and the individual countries with a baseline for systematic monitoring of availability of policies, from which aspects of implementation will be added. In the South-East Asia region, both country offices and the regional office have begun to use the data for decision-making, a trend that should hopefully continue throughout other regions. The Eastern Mediterranean region has developed thematic policy briefs based on the findings of the survey. Future country and/or regional policy dialogues can help to promote use of the results of the survey in informing policy and program development and in learning about use of policy data for program implementation and accountability. Lessons learned from the process and content of the 2018-2019 SRMNCAH policy survey can help to improve not only future rounds of this survey but also other initiatives to collect legal and policy data whether at global, national or sub-national level.

## Acknowledgements

 The 2018-2019 SRMNCAH policy survey was completed by respondents across 155 countries. We gratefully acknowledge these respondents. Special thanks to Gloria Diamond, Michalina Drejza and Neha Jhaveri for their work in cataloguing thousands of documents for the searchable repository. We would like to thank Bernadette Daelmans, Blerta Maliqi and Marcus Stahlhofer for their technical advice and guidance. We also acknowledge the funding support from the Bill and Melinda Gates Foundation.

## Ethical issues

 The 2018-2019 WHO SRMNCAH policy survey followed necessary WHO protocols for non-emergency, non-human-subject data collection.

## Competing interests

 Authors declare that they have no competing interests.

## Authors’ contributions

 Conception and design: TD, EK. Acquisition of data: GB, ZL, NF, SB, BBR, CAJ, DK, KS, and KD. Analysis and interpretation of data: AS, EK, LNH, LF, CAJ, RM, KS, JK, MRF, and TD. Drafting of the manuscript: EK and TD. Critical revision of the manuscript for important intellectual content: ZL, NF, SB, LNH, BBR, AS, LF, RM, CAJ, NR, KS, JK, MRF, TD, and EK. Administrative, technical, or material support: GB, CAJ, and NR. Supervision: CAJ and NR.

## Disclaimer

 The authors alone are responsible for the views expressed in this article and they do not necessarily represent the views, decisions or policies of the institutions with which they are affiliated.

## Funding

 This work was supported, in whole or in part, by the Bill & Melinda Gates Foundation [INV-009058]. Under the grant conditions of the Foundation, a Creative Commons Attribution 4.0 Generic License has already been assigned to the Author Accepted Manuscript version that might arise from this submission.
